# Nutritional risk screening—a cross-sectional study in a tertiary pediatric hospital

**DOI:** 10.1186/s41043-019-0166-4

**Published:** 2019-03-25

**Authors:** J. Tuokkola, J. Hilpi, K-L Kolho, H. Orell, L. Merras-Salmio

**Affiliations:** 1 Children’s Hospital, Helsinki University Hospital and University of Helsinki, P.O. Box 281, FI-00029 HUH Helsinki, Finland; 2 Clinical Nutrition Unit, Internal Medicine and Rehabilitation, Helsinki University Hospital and University of Helsinki, Helsinki, Finland; 3 Tampere University and Tampere University Hospital, Tampere, Finland

**Keywords:** Pediatric hospital inpatient, Malnutrition, Nutritional risk screening

## Abstract

**Background:**

All hospitalized patients should be screened for malnutrition risk. No universal method exists for pediatric patients.

**Methods:**

We performed a cross-sectional study comparing three published malnutrition risk screening tools (PYMS, STAMP, and STRONG_kids_), applying them to each inpatient aged 1 month to 17 years over a period of five consecutive weekdays in Helsinki University Hospital, Finland.

**Results:**

Of the eligible patients, 67% (*n* = 69) participated. We found that 6.2% of the children were acutely malnourished and accurately categorized by the three tools. STRONG_kids_ showed the highest specificity (100%) and positive predictive value (36%). Acute malnutrition seemed to be associated with longer hospital stay (*p* = 0.051).

**Conclusion:**

STRONG_kids_ was the most accurate screening tool for detecting acute malnutrition and was therefore chosen as the screening method in our hospital. Routine screening for the risk of malnutrition in pediatric inpatients is important in detecting at-risk children who would otherwise be left without dietary intervention.

**Electronic supplementary material:**

The online version of this article (10.1186/s41043-019-0166-4) contains supplementary material, which is available to authorized users.

## Introduction

Malnutrition is an underrecognized problem leading to an increased risk for complications, longer hospital stays, and decreased quality of life [1, 2]. Several guidelines suggest that pediatric hospital inpatients should be screened for malnutrition risk [3]. Consensus on which screening method is preferable in pediatric patients is lacking [4, 5].

Three validated bedside screening methods for malnutrition risk have been developed in recent years: The Screening Tool for the Assessment of Malnutrition in Paediatrics (STAMP) [6], the Pediatric Yorkhill Malnutrition Score (PYMS) [7], and the Screening Tool for Risk of Impaired Nutritional Status and Growth (STRONG_kids_) [8]. These screening methods share some similarities in questions regarding appetite, weight development, and underlying illness. The STAMP and STRONG_kids_ methods include a list of high-risk diagnoses. STAMP and PYMS, but not STRONG_kids,_require anthropometric measurements.

The screening tools aim to detect children whose outcome may improve with dietary intervention. The tools classify children as having low, medium, or high risk for malnutrition. Several studies in different countries and settings have compared the applicability of these tools [4, 6–15]. Smaller studies have favored the STRONG_kids_ screening tool over PYMS or STAMP due to its higher accuracy. However, a large European multicenter study [4] and a meta-analysis [5] do not support the use of one screening method over another. Thus, we sought to determine which of the three tools has the highest accuracy for use in daily practice.

## Methods

### Subjects

Inpatients aged 1 month to 17 years, staying for least 24 h in the pediatric or surgical wards of Helsinki University Children’s Hospital, Finland, were invited to participate in the study. We excluded patients treated in pediatric and neonatal intensive care units and those children whose families did not speak Finnish. The study period was five consecutive weekdays, Monday through Friday, once on each ward (*n* = 8) in October to November 2013. Information from the three screening methods was compiled into a set of questions, and the separate risk scores were drawn from the data (Additional file 1: Table SI).

### Data collection

Weight and height or recumbent length were recorded, and national growth charts were used to draw height and expected body mass index (BMI) standard deviation (SD) scores and weight-to-height ratio [16–18]. ISO-BMI is a BMI-for-age cut-off value for thinness, overweight, and obesity with the cut-off curves passing through specified BMIs at the age of 18 years. ISO-BMI values are defined for children from 2 years of age onwards. Malnutrition was defined according to the WHO guidelines plotted on the national growth charts with − 2 SD of weight-to-height or BMI (representing acute malnutrition) and − 2 SD of height-to-age (representing chronic malnutrition) as the cut-off point. Children’s diagnosis and length of hospital stay (LOS) were derived from hospital records. Parents answered a question on how worried they are on a scale from one to seven regarding their child’s nutrition. A single trained nutritionist (JH) conducted the data collection.

### Ethical issues

The study protocol was approved by the Ethics Committee of Helsinki University Hospital. All families provided written informed consent.

### Statistical analysis

SPSS software version 24 (IBM Corp., Armonk, NY, USA) was used for statistical analysis: Cohen’s kappa for agreement between nutrition risk tools, chi-square and Fisher’s exact tests for differences between categories, and Mann-Whitney *U* test and Kruskal-Wallis test for associations between LOS and malnutrition or risk scores. For the ability to detect acute malnutrition, positive and negative predictive values and sensitivity and specificity were calculated. Statistical significance was set at *p* < 0.05.

## Results

### Patients

Of the 103 eligible children, aged 1 month to 17 years (mean age 6.1 years), 69 (67%) participated: 33 girls and 36 boys (Table 1). The parents of 20 children could not be reached, and 14 families chose not to take part in the study. Median total LOS was 5 days (range 1–234 days).

**Table 1:** Prevalence of malnutrition in 69 inpatients aged 1 month to 17 years in tertiary pediatric hospital wards, excluding intensive care units

Assessment method	Number (%) of malnourished children
Weight-for-height according to national growth charts*	4/65** (6.2%)
ISO-BMI < 17*** (over 2-year-olds)	6/48 (12.5%)
Height-for-age < − 2 SD according to national growth charts	10/65** (15.3%)

*< − 15% for < 130 cm, < − 20% for 130–160 cm, and < − 25% for > 160 cm

**Height measurements were obtained for 65 patients

***Represents < − 2 SD, i.e., grade 2 thinness

*BMI* body mass index, *ISO-BMI* BMI-for-age cut-off value for thinness, overweight, and obesity

### Malnutrition risk screening

The PYMS tool classified the most (44%) and STRONG_kids_ the least (16%) children as having a high risk for malnutrition (Table 2). Cohen’s kappa between PYMS and STAMP was *κ* = 0.512, between PYMS and STRONG_kids_*κ* = 0.257, and between STAMP and STRONG_kids_*κ* = 0.309. STRONG_kids_ showed the highest specificity and positive predictive value for acute malnutrition (Table 2).

**Table 2:** Malnutrition risk scores and positive and negative predictive values and sensitivity and specificity of their ability to detect acute malnutrition in 69 inpatients aged 1 month to 17 years in tertiary hospital wards, excluding intensive care units

Malnutrition risk score	PYMS	STAMP	STRONG_kids_
Low, *n* (%)	18 (26.1%)	5 (7.2%)	12 (17.4%)
Medium, *n* (%)	21 (30.4%)	40 (58.0%)	46 (66.7%)
High, *n* (%)	30 (43.5%)	24 (34.8%)	11 (15.9%)
Sensitivity	100%	100%	100%
Specificity	60%	69%	89%
Negative predictive value	100%	100%	100%
Positive predictive value	13%	17%	36%

*PYMS* Pediatric Yorkhill Malnutrition Score, *STAMP* The Screening Tool for the Assessment of Malnutrition in Paediatrics, *STRONG*_*kids*_ the Screening Tool for Risk of Impaired Nutritional Status and Growth

Acute malnutrition defined as weight-for-height according to national growth charts < − 15% for < 130 cm, < − 20% for 130–160 cm, and < − 25% for > 160 cm

### Prevalence of malnutrition

Height and weight measurements were available for 65/69 children (94.2%). Of these, four (6.2%) were acutely malnourished. They were from cardiac, renal, oncologic, and psychiatric specialties; *p* = 0.497 for differences in the prevalence of malnutrition across the specialties. All three assessment tools classified these acutely malnourished children as having a high nutritional risk.

Of the 65 children, 10 (15.4%) had height below − 2 SD, suggestive of chronic malnutrition, although in six the likely explanation was either a syndrome affecting growth or use of corticosteroids. They were categorized as having medium or high risk by all three methods, except for one child, who was categorized as having a low risk by PYMS.

### Length of hospital stay

Children with a high risk for malnutrition stayed in hospital longer (Table 3). Median (interquartile range, IQR) LOS was also longer in acutely malnourished children than in those not acutely malnourished (17.5 days, 12–58 vs. 5 days, 2–13, respectively), (*p* = 0.051). The LOS for those whose height SD was below and above − 2 SD was 14 days (4–121) and 4.5 days [2–12], respectively (*p* = 0.090). Age of the child did not differ between malnourished or well-nourished groups or between the risk scores of any of the screening methods.

**Table 3:** Length of stay (days, interquartile ranges) in different nutrition risk categories according to three screening tools in 69 inpatients aged 1 month to 17 years in tertiary hospital wards, excluding intensive care units

Categories	Low	Medium	High	*p* value*
PYMS	2.5 (1–9)	4 (2–8)	12 (2–39)	0.038
STAMP	2 (1–4)	3.5 (2–9)	13.5 (4–51)	0.001
STRONG_kids_	4 (1–8)	4 (2–14)	12 (6–27)	0.134

*Kruskal-Wallis test

*PYMS* Pediatric Yorkhill Malnutrition Score, *STAMP* The Screening Tool for the Assessment of Malnutrition in Paediatrics, *STRONG*_*kids*_ the Screening Tool for Risk of Impaired Nutritional Status and Growth

### Dietetic contacts

Three of the four acutely malnourished patients had an ongoing contact either prior or during the present admission as well as active follow-up with a dietitian and one had had dietary counseling in the past (*p* = 0.194). Of those children with high nutritional risk scores with PYMS, 25 were with and 5 without dietetic contact (*p* = 0.001); with STAMP, 21 were with and 3 without dietetic contact (*p* < 0.001); and with STRONG_kids_, 10 were with and 1 without dietetic contact (*p* = 0.006). High parental concern for the child’s nutrition was associated with acute malnutrition (*p* = 0.042) as well as with high PYMS (*p* < 0.001), STAMP (*p* = 0.005), and STRONG_kids_ (*p* = 0.003) risk scores.

## Discussion

In our tertiary care pediatric hospital, 6.2% of children were acutely malnourished, comparable to the 7% reported in a large European multicenter study [1]. Depending on the screening method, one in 11 to one in six high-risk patients was without dietetic contact, and one in four acutely malnourished children. Without screening, they would have gone unnoticed. STRONG_kids_ showed the highest specificity and positive predictive value for acute malnutrition. Thus, based on these results and for ease of use, it will be used as the screening method in our hospital.

Ease and swiftness of use are crucial in implementing a screening tool for malnutrition in pediatric inpatients. Measuring the child’s height seems to be a major issue for nurses and is the reason cited for not completing a screening tool that requires anthropometrics [19, 20]. Screening with STRONG_kids_ takes less than 5 min and has been considered easy to understand by nurses [11]. As a research dietitian carried out our study, we could not determine nurses’ views on this issue.

The main strength of the study is that over a short period a single trained researcher performed all patient screenings, thus excluding inter-rater variation. Limitations of the study include the modest sample size and the lack of healthy controls. However, the patients included were representative of tertiary hospital pediatric and surgical ward inpatients. The number of patients was lower than expected because the number of patients in the wards during data gathering was lower than expected based on previous years’ statistics.

In accordance with our results, several other studies comparing screening methods have also favored STRONG_kids_ as a screening method because of its high specificity [9, 15]. However, a large European multicenter study [4] and a meta-analysis [5] do not support the use of one screening method over another due to insufficient evidence and because all methods may not identify children with subnormal anthropometric measurements. Nevertheless, defining malnutrition is more complex than mere anthropometrics [21]. Especially defining chronic malnutrition by height SD is problematic, as is highlighted by our findings that over half of those who were categorized as chronically malnourished by WHO standards had non-nutritional reasons for their stunting. This makes height SD a poor marker for malnutrition in developed countries, and a suboptimal reference for malnutrition screening methods.

Poor nutritional status and high nutritional risk scores have been correlated with increased LOS and treatment costs in several studies [1, 4, 8, 10, 13, 14]. The largest study assessing the relation between LOS and nutritional risk screening found coefficients ranging from 1.4 for PYMS and STAMP to 1.8 for STRONG_kids_ between low-risk and high-risk groups [4]. In our study, acute malnutrition was borderline significantly associated with longer hospital stay, while the difference in LOS between STRONG_kids_ risk categories (4 days in low- and medium-risk and 12 days in high-risk groups) was not statistically significant, possibly due to our small sample size. A recent meta-analysis stresses that the observed association between nutritional risk screening and LOS has not been shown to be causal [5].

In conclusion, it is important to recognize the children at risk of becoming malnourished. Early intervention preserves quality of life and diminishes costs and length of hospital stay. Bearing in mind the possible pitfalls, universal and easily applicable screening for malnutrition in inpatients is encouraged.

## Additional file

Additional file 1: **Table S1.** Questions in each of the screening methods: the Pediatric Yorkhill Malnutrition Score (PYMS), the Screening Tool for the Assessment of Malnutrition in Paediatrics (STAMP), and the Screening Tool for Risk of Impaired Nutritional Status and Growth (STRONG_kids_). (DOC 30 kb)

## Abbreviations

BMI: Body mass index
LOS: Length of stay
PYMS: Pediatric Yorkhill Malnutrition Score
SD: Standard deviation
STAMP: Screening Tool for the Assessment of Malnutrition in Paediatrics
STRONG_kids_: Screening Tool for Risk of Impaired Nutritional Status and Growth
