# Supplementary material for: Nutritional risk screening—a cross-sectional study in a tertiary pediatric hospital
Source: J Health Popul Nutr. 2019 Mar 25;38:8. doi: 10.1186/s41043-019-0166-4 (PMC6432750; doi:10.1186/s41043-019-0166-4)
Supplement: Supplementary file 1 — Table S1. Questions in each of the screening methods: the Pediatric Yorkhill Malnutrition Score (PYMS), the Screening Tool for the Assessment of Malnutrition in Paediatrics (STAMP), and the Screening Tool for Risk of Impaired Nutritional Status and Growth (STRONGkids). (DOC 30 kb) [file 41043_2019_166_MOESM1_ESM.doc]

Table SI. Questions in each of the screening methods: the Pediatric Yorkhill Malnutrition Score (PYMS), the Screening Tool for the Assessment of Malnutrition in Paediatrics (STAMP) and the Screening Tool for Risk of Impaired Nutritional Status and Growth (STRONGkids).

| PYMS | Is the BMI below the cutoff value shown in the BMI scoring guide?  Has the child lost weight recently?  Has the child had reduced intake (including feeds) for at least the past week (usual intake / reduced intake / no intake)?  Will the child’s nutrition be affected by the recent admission or condition for at least the next week? |
| --- | --- |
| STAMP | Use a growth chart or the percentile quick-reference tables to determine the child’s weight and height measurements.  What is the child’s nutritional intake (none / recently decreased or poor / no change or good)?  Does the child have a diagnosis that has any nutritional implication (definitely / possibly / no; list of diagnosis included in the instructions)? |
| STRONGKIDS | Is the patient in a poor nutritional status judged by a subjective clinical assessment?  Is there weight loss (infants) or poor weight gain (1 year olds and older) during the last few weeks or months?  Is one of the following items present: excessive diarrhoea (≥5 times/d) or vomiting (>3 times/d), reduced food intake during the last few days, pre-existing nutritional intervention, or inadequate nutritional intake because of pain?  Is there an underlying illness with risk of malnutrition or expected major surgery? (List of diagnosis included in the instructions). |
